# Differential sensitivity of prostate tumor derived endothelial cells to sorafenib and sunitinib

**DOI:** 10.1186/1471-2407-14-939

**Published:** 2014-12-12

**Authors:** Alessandra Fiorio Pla, Alessia Brossa, Michela Bernardini, Tullio Genova, Guillaume Grolez, Arnaud Villers, Xavier Leroy, Natalia Prevarskaya, Dimitra Gkika, Benedetta Bussolati

**Affiliations:** Department of Molecular Biotechnology and Health Sciences, Molecular Biotechnology Centre, University of Torino, via Nizza 52, 10126 Torino, Italy; Department of Life Science and Systems Biology, University of Torino, Torino, Italy; Nanostructured Interfaces and Surfaces Centre of Excellence (NIS), University of Turin, Torino, Italy; Inserm U1003, Equipe labellisée par la Ligue Nationale contre le cancer, Université des Sciences et Technologies de Lille (USTL), Villeneuve d’Ascq, France; Laboratory of Excellence, Ion Channels Science and Therapeutics, Université de Lille 1, Villeneuve d’Ascq, France; Department of Urology, CHU Lille, University Lille Nord de France, F-59000 Lille, France; Institute of Pathology, Centre de Biologie-Pathologie, CHRU de Lille, Faculté de Médecine Henri-Warembourg, Université de Lille 2, Lille, France

**Keywords:** Anti-angiogenic therapy, VEGF receptor, Androgen receptor, Prostate cancer, Drug resistance

## Abstract

**Background:**

Prostate cancer is the second leading cause of male cancer death in developed countries. Although the role of angiogenesis in its progression is well established, the efficacy of anti-angiogenic therapy is not clearly proved. Whether this could depend on differential responses between tumor and normal endothelial cells has not been tested.

**Methods:**

We isolated and characterized three lines of endothelial cells from prostate cancer and we tested the effect of Sunitinib and Sorafenib, and the combined treatment with the anti-androgen Casodex, on their angiogenic functions.

**Results:**

Endothelial cells isolated from prostate tumors showed angiogenic properties and expression of androgen and vascular endothelial cell growth factor receptors. Sunitinib affected their proliferation, survival and motility while Sorafenib only showed a minor effect. At variance, Sunitinib and Sorafenib showed similar cytotoxic and anti-angiogenic effects on normal endothelial cells. Sorafenib and Sunitinib inhibited vascular endothelial cell growth factor receptor2 phosphorylation of prostate cancer endothelial cells, while they differentially modulated Akt phosphorylation as no inhibitory effect of Sorafenib was observed on Akt activation. The combined treatment of Casodex reverted the observed resistance to Sorafenib both on cell viability and on Akt activation, whereas it did not modify the response to Sunitinib.

**Conclusions:**

Our study demonstrates a resistant behavior of endothelial cells isolated from prostate cancer to Sorafenib, but not Sunitinib. Moreover, it shows the benefit of a multi-target therapy combining anti-angiogenic and anti-hormonal drugs to overcome resistance.

**Electronic supplementary material:**

The online version of this article (doi:10.1186/1471-2407-14-939) contains supplementary material, which is available to authorized users.

## Background

Prostate cancer is one of the most common malignancies and remains the second leading cause of cancer death in men [1]. The improved understanding of prostate cancer biology in recent years led to the development of drugs directed against precise tumorigenesis-associated molecular pathways [2]. Angiogenesis, the development of new blood vessels, is recognized as one of the hallmarks of malignancy and prostate vasculature has been shown to play an important role in regulating the size and function of prostate malignancies [3, 4]. Accordingly, several anti-angiogenic drugs have been tested in phase II and III trials in prostate cancer patients [5], including the oral non-selective tyrosine kinase inhibitors Sunitinib and Sorafenib. These two drugs share their activity on vascular endothelial growth factor-receptors (VEGFRs), platelet derived growth factor-receptor beta (PDGFRβ), cKIT and RET, expressed on the cell membrane. In addition, Sorafenib is also able to directly act on the RAF intracellular pathway [6].

A bulk of evidence indicates that tumor blood vessels differ significantly from normal vessels for the structural organization and for the properties of endothelium [7–12]. This suggests that tumor vascularization depends on mechanisms alternative to the simple recruitment from adjacent tissue of pre-existing blood vessels [13]. The most remarkable abnormality reported for tumor-derived endothelial cells (TEC) is the chromosomal instability [9]. In addition, serial analysis of gene expression showed that TEC express genes not shared by blood vessels that reside in normal tissues [11]. Embryonic genes are expressed also by the endothelial cells derived from tumors [10, 14, 15]. Finally, TEC present functional alterations linked to increased survival, proliferation and angiogenic properties [13], as well as resistance to chemotherapeutics [16]. All these molecular and functional alterations in TEC may result in altered sensitivity to the anti-angiogenic therapy. However, information on the phenotype of TEC derived from prostate tumor and on their sensitivity to anti-angiogenic drugs is limited [17].

In the present study, we isolated and characterized three lines of TEC from three different prostate cancer human samples (PTEC). Moreover, we evaluated the effect of two anti-angiogenic drugs, Sunitinib and Sorafenib, on typical aspects of the angiogenic process such as the ability to form functional blood vessels *in vivo*, *in vitro* proliferation, survival, tubulogenesis and motility. Finally, as androgen receptor (AR) stimulation was reported to promote endothelial cell proliferation, we explored the possible effect of a combined treatment with anti-androgen and anti-angiogenic drugs.

## Methods

### Cancer tissue sampling

Prostate tissue samples (prostate adenocarcinoma) were obtained by Prof. Arnauld Villers and Prof Xavier Leroy (Dept. of Urology, Regional University Hospital of Lille, France) from 3 patients with a mean age of 58 years (ranging from 57 to 59) who underwent radical prostatectomy. Immediately after prostate removal (delay < 10 min), small pieces of tissue (at least 6 tissue samples from 0.5 to 1 cc) were grossly dissected by the pathologist from the left area, the right peripheral zone and the transitional area. To ensure tissue was malignant and to confirm the Gleason score, histological analysis of sections was performed on each sample by the same pathologist (Table 1). Patient verbal and written information and signed consent form required by the tissue collection unit by law was performed and obtained for all patients. This study was in accordance with the ethical requirements of the tissue collection unit of the Centre Hospitalier Régional Universitaire de Lille, University Lille Nord de France.

**Table 1:** **Characteristics of the patients used for the isolation of PTEC**

Patient	Age	Gleason score	Stage	PSA (ng/ml)	Androgen ablation
**01**	57	9 (4 + 5)	pT3b N0 M0	7.88	NO
**02**	59	9 (4 + 5)	pT3a N0 M0	11.46	NO
**03**	58	7 (3 + 4)	pT3 N0 M0	8.03	NO

### Drugs

Sunitinib malate (Sigma-Aldrich, St Louis, MO, USA), was resuspended in DMSO to a final concentration of 10 mM and stored at +4°C, according to the manufacturer’s instructions. Sorafenib (Bayer Pharmaceuticals, Leverkusen, Germany) was resuspended in DMSO to a stock concentration of 10 mM and stored at -20°C. Bicalutamide (Casodex) (Sigma-Aldrich, St Louis, MO, USA), was resuspended in DMSO to a stock concentration of 10 mM, according to the manufacturer’s instructions. Drugs were diluted into the culture medium shortly before performing the assays.

### Isolation of PTEC and other cell types

Prostate tumor endothelial cells (PTEC) were isolated on the basis of endothelial-specific culture conditions. For the isolation of PTEC, specimens were finely minced and digested in RPMI (Lonza, Basel, Switzerland) containing Collagenase IV (Sigma-Aldrich, St Louis, MO, USA) for 30 minutes at 37°C. After washings in medium plus 10% fetal calf serum (FCS, Seromed, Poly-Labo), the cell suspension was forced through a graded series of meshes to separate the cell components from stroma and aggregates. Cells (2×10^4^/cm^2^) where then plated in ECAF (Endothelial Cells Attachment Factor, Sigma-Aldrich, St Louis, MO, USA)-coated plates in EndoGRO MV-VEGF medium (Merck-Millipore, Billerica, Massachusetts, USA) containing 5% FCS, and maintained in culture for at least 6 passages. To avoid a possible fibroblast contamination, cells were cultured at passage one for three days with D-valine-substituted DMEM (Sigma-Aldrich, St Louis, MO, USA). Breast tumor endothelial cells (BTEC) were isolated and characterized as previously described [18]. Human umbilical vein endothelial cells (HUVEC) and microvascular endothelial cells (HMEC) were obtained from the umbilical vein or from derma, respectively, as previously described [19]. All endothelial cells were maintained in culture in EndoGRO MV-VEGF medium containing 5% FCS.

Human Embryonic Kidney (HEK) 293 and Lymph Node Carcinoma of Prostate (LNCaP) C4- 2 cells were grown in DMEM and RPMI 1684 (Invitrogen), respectively, supplemented with 10% FCS, L-glutamine (5 mM) (Sigma-Aldrich, St Louis, MO, USA) and kanamycin (100 mg/ml) (Sigma-Aldrich, St Louis, MO, USA). Cells were transfected with 2 μg of pcDNA4-AR construct using FuGENE HD reagent (Roche Diagnostics, France), as described [20].

### Flow cytofluorimetric and immunofluorescence analysis

For cytofluorimetric analysis, PTEC lines were detached from plates with a non-enzymatic cell dissociation solution (Sigma-Aldrich, St Louis, MO, USA), washed and stained (30 min at 4°C) with the following fluorescein isothiocyanate (FITC)-, phycoerythrin (PE)-, or allophtcocyanin (APC)-conjugated antibodies: PDGFRβ, CD31 (all from BD Bioscience, Franklin Lakes, NJ, USA) CD105, VEGR2 (all from MiltenyiBiotec, Bergisch Gladbach, Germany), c-KIT (Dako, Glostrup, Denmark), TIE2, VEGFR1, VEGFR3 (all from R&D Systems, Minneapolis, MN, USA). Isotypes (all from MiltenyiBiotec, Bergisch Gladbach, Germany) were used as negative controls. Cells were subjected to cytofluorimetric analysis (FACScan Becton Dickinson, Franklin Lakes, NJ, USA) at each culture passage. Indirect immunofluorescence was performed on cells cultured on chamber slides (Nunc, Roskilde, Denmark). Cells were fixed in 3.5% paraformaldehyde containing 2% sucrose and permeabilized with Hepes-Triton X-100 0.1% for 10 minutes at 4°C. The anti-pan-cytokeratin polyclonal Ab (Biomeda, Foster City, California, USA) was used. Texas Red goat anti-rabbit IgG (Molecular Probes, Eugene, OR, USA) was used as secondary antibody. Hoechst 33258 dye (Sigma-Aldrich, St Louis, MO, USA) was added for nuclear staining. Confocal microscopy analysis was performed using a Zeiss LSM 5 Pascal model confocal microscope (Carl Zeiss, Oberkochen, Germany).

### *In vitro*tubule formation

*In vitro* formation of capillary-like structures was studied on growth factor-reduced Matrigel (BD Bioscience, Franklin Lakes, NJ, USA) in 24-well plates. PTEC, HUVEC and HMEC (3,5 × 10^4^ cells/well) were seeded onto Matrigel-coating in EndoGRO MV-VEGF medium containing 5% FCS and treatments performed in duplicate. Cell organization onto Matrigel was periodically imaged with a Nikon Eclipse Ti inverted microscope using a Nikon Plan 10X/0,10 objective and cells were kept on a stage incubator at 37°C and 5% CO2 during the experiment (OKOLab, Italy). Images were acquired at 2 h time intervals using MetaMorph software.

Image analysis was performed with ImageJ software: images at 18 hours of treatment were analyzed: number of nodes (intersections formed by at least three detectable cells) and total tubule length (aligned cells connecting nodes) were measured for each field. Number of nodes and tubule length were normalized to maximum values and their sum for each condition was used to express the grade of organization in "capillary-like" structures in terms of arbitrary units (A.U.). At least ten fields for each condition were analyzed in each independent experiment. Graphs show mean values of three independent experiments, error bars show standard error. Values are expressed as mean ± S.E.M.

### *In vivo*tubule formation

To evaluate the tubule formation *in vivo*, 2 × 10^6^ PTEC were implanted subcutaneously into SCID mice (Charles River, Wilmington, Massachusetts) within growth factor–reduced Matrigel (BD Biosciences, Franklin Lakes, NJ, USA) as previously described [10]. Briefly, cells were harvested and resuspended in 150 μl DMEM plus 250 μl of Matrigel, chilled on ice and injected subcutaneously into the left back of SCID mice (n = 4). After 7 days, mice were sacrificed, and endothelial plugs recovered and processed for histology. Typically, the overlying skin was removed, and gels were cut out by retaining the peritoneal lining for support, fixed in 10% buffered formalin, and embedded in paraffin. Sections (3 μm) were cut and stained with hematoxylin and eosin and were examined under a light microscope system.

### Proliferation assay and MTT

For the cytotoxicity or the proliferation assay, cells were plated in the growth medium at a concentration of 3000 cells/well in a 96-multiwell plate and left in adhesion overnight. The day after the culture medium was removed and cells were incubated with Sunitinib or Sorafenib in RPMI 2% FCS. After 48 h, DNA synthesis was detected after as incorporation of 5-bromo-2-deoxyuridine (BrdU) using an enzyme-linked immunosorbent assay kit (Roche, Penzberg, Germany). Cytotoxicity was evaluated by MTT (3-(4,5-dimetiltiazol-2-yl)-2,5-difenil tetrasodium bromide) (Merck-Millipore, Billerica, Massachusetts, USA), according to manufacturer’s instructions. Data are expressed as the mean ± S.E.M of the media of absorbance of at least three different experiments performed with all the three lines in the study in triplicate, normalized to the positive control (vehicle alone). To evaluate the IC_50_ of both Sunitinib and Sorafenib on HUVEC, MTT data were analysed using Calcusyn software. Results are expressed as mean ± S.E.M. of five different experiments.

### Cell migration

Migration was assessed using silicone culture inserts (ibidi GmbH, Munich, Germany) in 12-well culture plates. Inserts had two 70 μl wells, both of which were used to plate cells. PTEC, HUVEC or BTEC (1 × 10^5^cells/ml) were plated on 1% gelatin coating in EndoGRO MV-VEGF medium containing 5% FCS. Cells were maintained in incubator until confluence was reached. Cell monolayers were starved 12 hours in DMEM 0% FCS before removing the inserts and thus generating the "wound area". Floating cells were removed by wash in PBS solution, and monolayers were treated with test conditions (in duplicate). EndoGRO MV-VEGF medium 5% FCS was used as positive control, whereas DMEM 0% FCS served as negative control. Cell migration was imaged with a Nikon Eclipse Ti inverted microscope using a Nikon Plan 4X/0, objective and cells were kept on a stage incubator at 37°C and 5% CO2 during the experiment (OKOLab, Italy). Images were acquired at 2 h time intervals using MetaMorph software.

MetaMorph software was used to calculate migration rate (%) by measuring the distance covered by cells between two subsequent time points (4 fields measurements for each image). Measurements were made for each time point and at least 10 fields for each condition were analyzed in each independent experiment. Graphs show mean ± S.E.M of three independent experiments.

### Western blot analysis

PTEC and HUVEC were grown in ENDOGRO MV-VEGF medium containing 5 and 10% FCS respectively, until cells reached confluence. Cells were then incubated 10 or 30’ with vehicle, Sunitinib or Sorafenib (1 and 2.5 μM) and lysed at 4°C for 30 min in RIPA buffer (20 nMTrisHCl, 150 nMNaCl, 1% deoxycholate, 0.1% SDS, 1% Triton X-100, pH 7.8) supplemented with protease inhibitor cocktail, PMSF (all from Sigma-Aldrich, St Louis, MO, USA) and PhosStop (Roche, Penzberg, Germany). Conditions for Western blotting were as described previously [21]. Polyvinylidene fluoride membranes were blocked and incubated overnight with goat polyclonal anti VEGFR2 (R&D Systems, Minneapolis, MN, USA) antibody (1:2000); or rabbit polyclonal anti Phospho-VEGFR2-Tyr951 (p-VEGFR2; sc-101821, Santa Cruz Biotechnology, Santa Cruz, CA, USA) antibody (1:100); rabbit polyclonal anti p44/42MAPK (ERK1/2) (9102, Cell Signalling, Danvers, MA, USA) antibody (1:1000); rabbit polyclonal anti Phospho-p44/42 MAPK (pERK1/2) (Thr202/Tyr204) (4370, Cell Signalling, Danvers, MA, USA) antibody (1:1000); rabbit polyclonal anti Akt (9272, Cell Signalling) antibody (1:3000); rabbit monoclonal IgG Phospho-Akt (pAkt; Ser473) (4508, Cell Signalling) antibody (1:2000); rabbit polyclonal anti AR (N-20) antibody (1:400) (sc-816, Santa Cruz Biotechnology). Membranes were then washed with 1X TBST containing 0.1% Tween 20 and incubated as required with the HRP-conjugated anti-goat (Dako, Glostrup, Denmark) or anti-rabbit IgG (Santa Cruz Biotechnology) antibodies. Chemiluminescence detection was conducted using the ECL prime Western blotting detection reagent (GE Healthcare, Buckinghamshire, England). To quantify the differences in protein phosphorylation, the ratio between non phosphorylated and phosphorylated protein expression was evaluated. Membranes were then washed with 1X TBST containing 0.1% Tween 20 and incubated as required with the HRP-conjugated anti-goat (Dako, Glostrup, Denmark) or anti-rabbit IgG (Santa Cruz Biotechnology) antibodies.

### RNA isolation and real time PCR

Total RNA was isolated using Trizol Reagent (Ambion, Life Technologies, Carlsbad, California, USA) according to the manufacturer’s protocol, and quantified spectrophotometrically (Nanodrop ND-1000). For gene expression analysis, quantitative real-time PCR was performed. Briefly, first-strand cDNA was produced from 200 ng of total RNA using the High Capacity cDNA Reverse Transcription Kit (Applied Biosystems, Foster City, California, USA).

Quantitative Real-time PCR experiments were performed in 20-μl reaction mixture containing 5 ng of cDNA template, the sequence-specific oligonucleotide primers (purchased from MWG-Biotech, Gmbh, Eurofins Genomics, Hamburg, Germany) and the Power SYBR Green PCR Master Mix (Applied Biosystems). 18S was used to normalize RNA inputs. Fold change expression respect to HMEC was calculated for all samples. The sequence-specific oligonucleotide primers used are: AR (NM_000044.3) forward, 5′-GCAGGAAGCAGTATCCGAAG-3′ (position 1709); reverse, 5′-CTCTCGCCTTCTAGCCCTTT-3′ (position 2067); 18S ribosomal RNA (18S, X03205) forward, 5′- CAGCTTCCGGGAAACCAAAGTC-3′ (position 1132); reverse, 5′- AATTAAGCCGCAGGCTCCACTC -3′ (position 1222). Primer amplification efficiencies were 100.2% for AR and 101.3% for 18S, and the slope values were -3.316 for AR and -3.291 for 18 s respectively. The comparative Ct method was adopted for relative quantification of gene expression and 18 s was used to normalize RNA inputs. Fold change expression respect to HMEC was calculated for all samples.

### Statistical analysis

Data are presented as means ± S.E.M. Statistical and significant differences were determined using one-way ANOVA with Newmann-Keuls or Dunnett multicomparison tests (GraphPad Prism version 4.00, GraphPad Software, San Diego, CA) or nonparametric unpaired Wilcoxon-Mann–Whitney test, as appropriate. A p value of < 0.05 was considered significant. Kolmogorov-Smirnov statistical analysis was used to test significant differences in cytofluorimetric data.

## Results

### Characterization of PTEC lines

**Figure 1:**
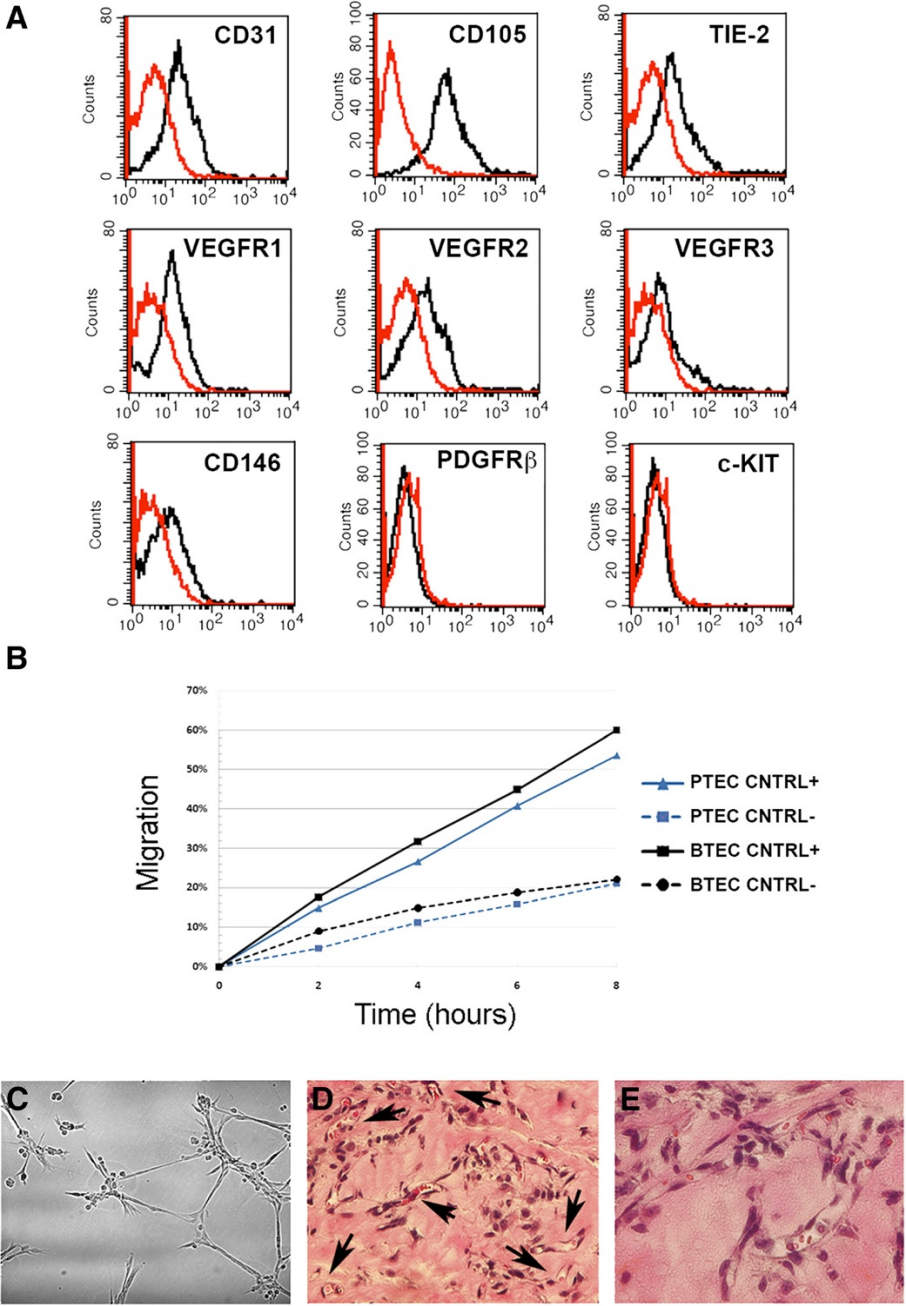
**Characterization of PTEC lines. (A)**: Representative cytofluorimetric analysis of a PTEC line at the first passage. Specific antibodies are shown as black line, isotypic controls as red line. **(B)**: Percentage of migration of PTEC (blue line) compared to endothelial tumor cells isolated from breast cancer (BTEC, black line) in EndoGRO plus 5% FCS (CNTRL+) or in DMEM without FCS (CNTRL-). **(C-E)**: Representative images showing PTEC organization *in vitro* and *in vivo. In vitro*, PTEC plated on Matrigel-coated wells organized in capillary-like structures (18 h) **(C)**. When injected in SCID mice within Matrigel, PTEC, organized in functional vessels containing red blood cells (arrows) (**D** and **E**, hematoxylin and eosin staining). Original magnification D: × 200, E: × 400. All the isolated lines showed a similar marker expression and functional properties *in vitro* and *in vivo*.

Endothelial cells were purified by prostate carcinomas of three patients who underwent radical prostatectomy (Table 2). Three cell preparations of PTEC (PTEC 1, 2, 3) were obtained from different tumors. Cells were characterized by cytofluorimetric analysis on the basis of positive expression of a panel of endothelial markers, such as CD31, CD105 and the angiopoietin receptor TIE-2 (Figure 1A and Table 2). PTEC expressed VEGFR1 and VEGFR2 but low levels of VEGFR3 (Figure 1), the lymphatic-associated VEGF receptor. Moreover, CD146 was also expressed at low levels (Figure 1), as previous reported on murine PTEC isolated from spontaneous prostate tumors [17]. The expression of endothelial markers was tested every culture passages and remained constant during cell culture for the three cell lines (Table 2). No cytokeratin positive cells were detected by immunofluorescence analysis (not shown).

The endothelial nature of PTEC was also showed by functional characteristics. PTEC display a migration rate comparable to that of breast tumor-derived ECs (BTEC) both in serum-free conditions and in endothelial medium (EndoGRO 5% FCS), as detected in wound healing assays by time-lapse microscopy (Figure 1B). Moreover, PTEC were able to organize in pre capillary-like structures onto Matrigel and to form tubules within 18 hours from seeding (Figure 1C). To evaluate the behavior of PTEC in vivo, cells (passage 2–4) were injected subcutaneously within diluted Matrigel in SCID mice. After 7 days, plugs were recovered and processed for histological analysis. All cell lines grew and spontaneously organized within 1 week in functional microvessels, connected to the mouse vasculature, as shown by the presence of blood cells and leukocytes (Figure 1C). Together, these data indicate that PTEC presents both phenotypical as well as functional properties of endothelial cells.

**Table 2:** **Expression of different endothelial markers by the three cell lines isolated in the study**

	***Cell lines***			
	PTEC1	PTEC2	PTEC3	AVERAGE
**Isotype**	5.32 ± 0.57	6.34 ± 0.77	5.69 ± 0.98	5.783 ± 0.83
**CD146**	6.598 ± 1.08	12.203 ± 2.87	14.727 ± 9.89	11.176 ± 4.93
**CD105**	91.614 ± 28.56	70.547 ± 17.45	65.707 ± 26.94	75.956 ± 19.62
**CD31**	27.942 ± 3.75	30.723 ± 4.46	24.54 ± 7.92	28.068 ± 5.89
**TIE-2**	23.555 ± 2.46	19.973 ± 4.77	29.13 ± 4.04	24.219 ± 4.84
**VEGFR1**	19.954 ± 5.35	8.68 ± 4.67	12.17 ± 2.78	13.575 ± 5.94
**VEGFR2**	20.782 ± 3.07	18.545 ± 3.71	25.83 ± 17.55	21.719 ± 8.32
**VEGFR3**	5.057 ± 2.69	7.811 ± 2.44	10.603 ± 3.92	7.823 ± 3.16

Levels of endothelial markers was detected by cytofluorimetric analysis after cell staining with a specific conjugated Abs. An irrelevant isotypic Ab (isotype) was used as control of aspecific binding. Data represent the median fluorescent intensity (MFI ± SD) detected on the cell isolates at all culture passages used (1–6). All markers were significantly different versus isotypic control (p < 0.001), as evaluated using the Kolmogorov-Smirnov statistical analysis.

### Effect of Sunitinib and Sorafenib on PTEC proliferation, cytotoxicity, tubulogenesis and migration

**Figure 2:**
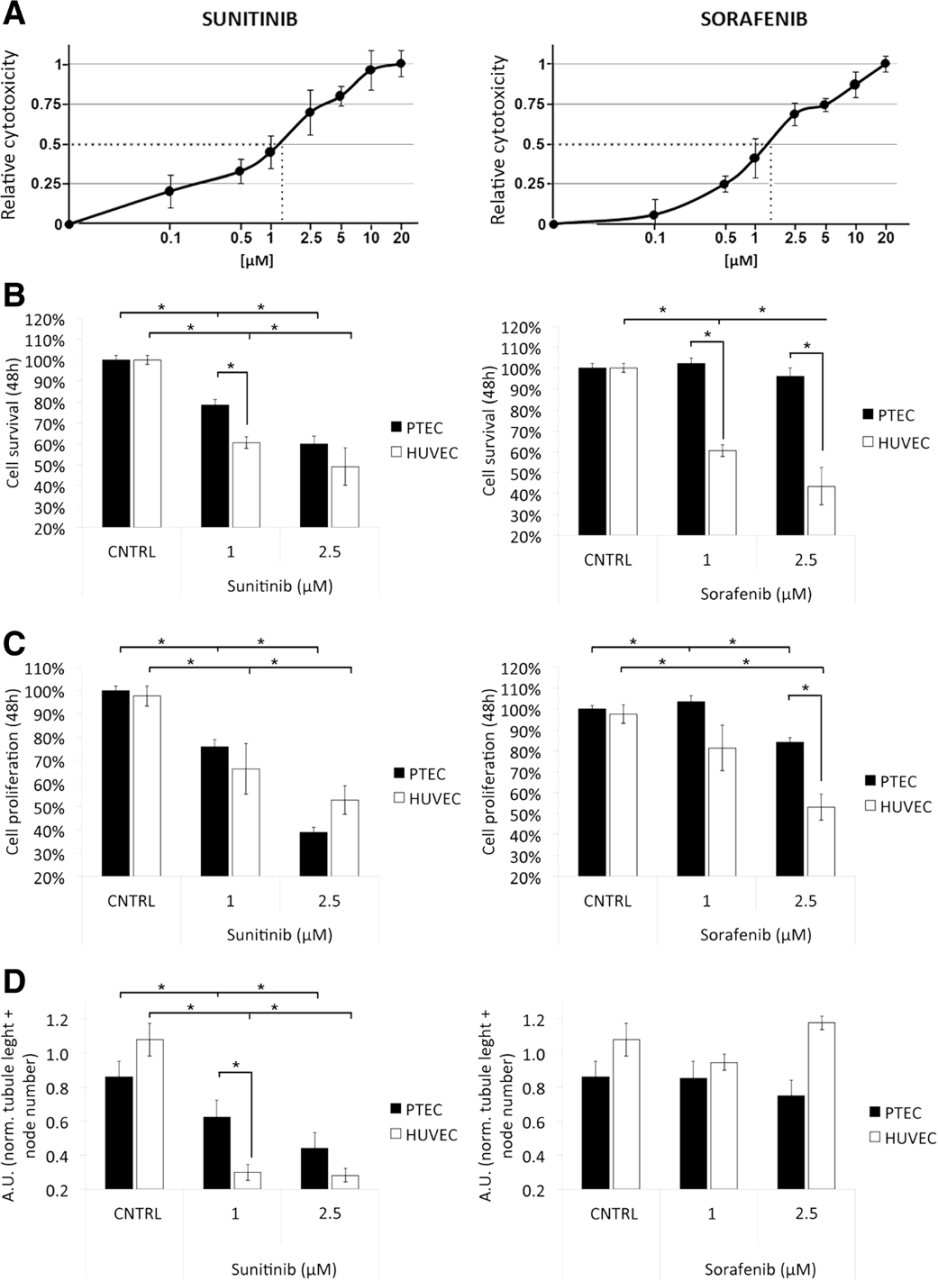
**Cytotoxicity resistance, proliferation and tubulogenesis of Sunitinib and Sorafenib treated PTEC and HUVEC. (A)**: Relative vitality of HUVEC treated with different concentrations (x-axis, logarithmic scale) of Sunitinib and Sorafenib, showing the IC_50_ (1.4675 μM for Sunitinib and 1.5329 μM for Sorafenib, dotted line). **(B** and **C)**: Cell survival and proliferation of PTEC (black columns) and HUVEC (white columns) after 48 h incubation with 1 μM and 2.5 μM Sunitinib or Sorafenib. Cytotoxicity was detected as MTT assay, proliferation as BrdU assay. **(D)**: Capillary-like organization of PTEC (black columns) and HUVEC (white columns). Cells were seeded on Matrigel and observed at different times points. Images at 18 hours of treatment were analyzed and total tubule length was measured for each field. Data are the mean ± S.E.M. of a minimum of three independent experiments performed with three (PTEC1, 2 and 3) or five (HUVEC) different cell lines in triplicate. Statistical significance *p < 0.05.

We subsequently evaluated the effect of Sunitinib and Sorafenib, two anti-angiogenic drugs currently in clinical trial for prostate cancer [5, 22–24] on PTEC functional properties. Both drugs impaired proliferation of normal endothelial cells with an IC_50_ at similar doses around 1.5 μM (1.4675 and 1.5329 μM respectively), as evaluated by MTT analysis (Figure 2A). PTEC were treated with 1 and 2.5 μM of both drugs. Sunitinib impaired survival and proliferation of PTEC at a concentration as low as 1 μM. At variance with Sunitinib, Sorafenib (1 μM) had no effect on both proliferation and survival of PTEC while a cytotoxic effect was observed at 2.5 μM (Figure 2B and C). On the other hand, both macrovascular (HUVEC) and microvascular (HMEC) endothelial cells, used as control, showed high sensitivity to both Sorafenib and Sunitinib (Figure 2B and C and Additional file 1: Figure S1 A and B).

We also studied the effect of Sunitinib and Sorafenib on PTEC organization in pre capillary-like structures onto Matrigel. As shown in Figure 2D, the ability of PTEC to organize in tube structures was strongly inhibited by Sunitinib, both a 1 μM and 2.5 μM at a similar extent to HUVEC and HMEC (Figure 2D and Additional file 1: Figure S1 C). In contrast, Sorafenib had no inhibitory effect on tubulogenesis both on PTEC and HUVEC, even at the higher dose tested (Figure 2D) and a minor effect on HMEC at higher doses (Additional file 1: Figure S1 C).

**Figure 3:**
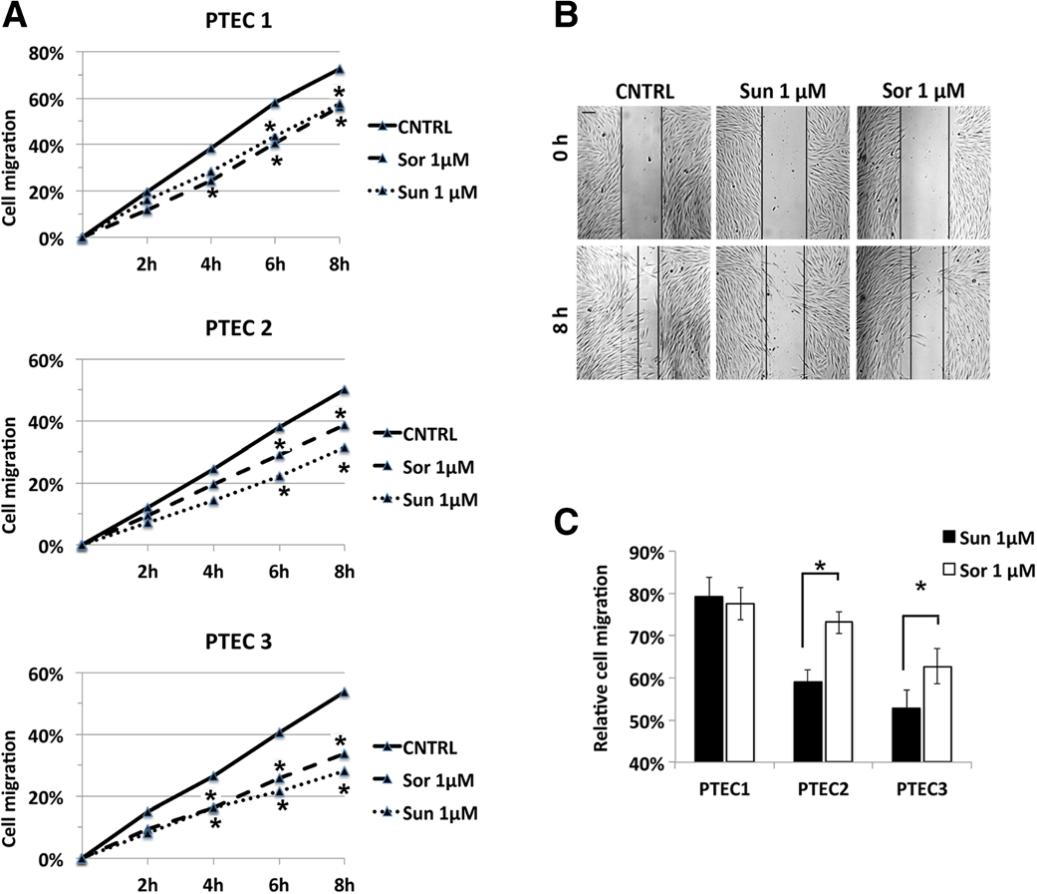
**Wound healing migration in PTEC lines. (A)**: percentage of migration of PTEC after treatment with 1 μM Sunitinib or 1 μM Sorafenib compared to positive control. **(B)**: representative images of PTEC migration at time 0 and 8 hours. **(C)**: quantification of relative cell migration at 8 hours for the three PTEC cell lines. 1 μM Sunitinib showed a higher inhibition of cell migration compared to 1 μM Sorafenib. Data are expressed as mean ± S.E.M of three independent experiments performed with PTEC1, 2 and 3 and migration was normalized to control: Statistical significance *p < 0.05.

The effect of Sorafenib and Sunitinib on PTEC was also tested in wound healing migration assays at the non-cytotoxic dose (1 μM). Figure 3A shows that both 1 μM Sunitinib and 1 μM Sorafenib significantly decrease cell migration of about 15-20% compared to control conditions starting from 4 to 6 hours after treatment in all of the three lines. However, 1 μM Sorafenib has significantly less effect than 1 μM Sunitinib in two cell lines out of three (Figure 3B and C), in line with the resistant behavior of PTEC showed in the previous biological assays.

### Sunitinib and Sorafenib reduce VEGFR2 phosphorylation

**Figure 4:**
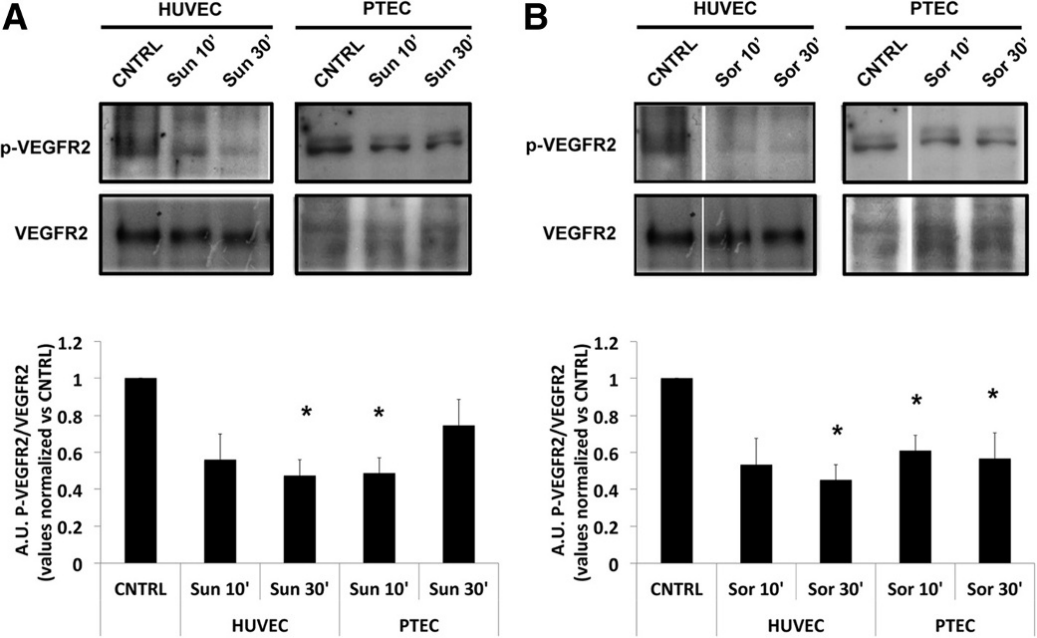
**Effect of Sorafenib and Sunitinib on VEGFR phosphorylation.** Western blot analysis showing basal VEGFR2 and phosphorylated p-VEGFR2(Tyr951) in PTEC and HUVEC after 10’ or 30’ treatment with 1 μM Sunitinib **(A)** and 1 μM Sorafenib **(B)**. The relative expression of p-VEGFR2(Tyr951) was normalized to basal VEGFR2. Values are expressed as mean ± S.E.M. relative to the control of three independent experiments performed with three (PTEC1, 2 and 3) and two (HUVEC) different cell lines. Statistical significance *p < 0.05.

PTEC expressed the VEGFRs, known to be target of anti-angiogenic tyrosine-kinases inhibitors Sunitinib and Sorafenib (Figure 1A) whereas they did not express other known targets such as the PDGFRβ and cKIT (Figure 1A), suggesting VEGFR2 as a main extracellular target for both Sorafenib and Sunitinib. In order to investigate whether the different sensitivity to Sorafenib observed for PTEC was due to a reduced inhibition of the main target, we evaluated VEGFR2 activity by Western blot analyses in the presence of the two anti-angiogenic drugs. Sunitinib or Sorafenib treatment (1 μM, 10 or 30 minutes) significantly reduced one of the major sites for VEGFR2 phosphorylation sites (Tyr951) [25] in both PTEC and HUVEC, used as control (Figure 4A). In particular, p-VEGFR2(Tyr951) levels decreased as soon as 10 minutes after treatment in PTEC, while in HUVEC the decrease was evident only at 30 minutes after treatment. Similarly, cell treatment with 1 μM Sorafenib decreased the relative expression of p-VEGFR2(Tyr951) both in PTEC and HUVEC after 10 or 30 minutes of treatment respectively (Figure 4B). The data therefore indicate that VEGFR2-activated signaling pathway is impaired by both Sunitinib and Sorafenib in PTEC at a similar extent as in HUVEC.

### Combination of anti-androgen and anti-angiogenic drugs

**Figure 5:**
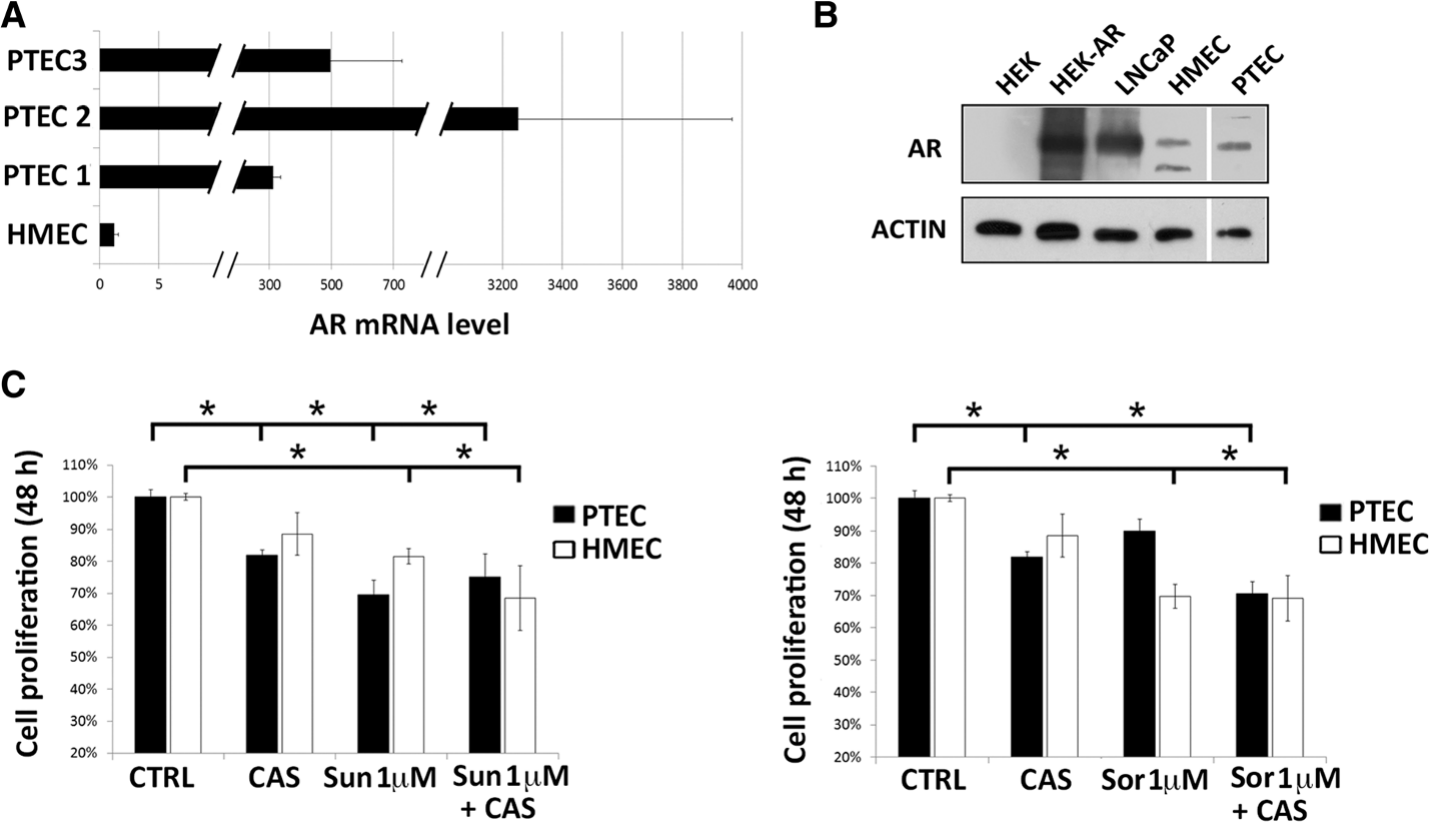
**Androgen inhibition impairs Sorafenib resistance in PTEC. (A)**: Analysis of Androgen receptor (AR) mRNA expression levels by qPCR in PTEC cell lines. Data were normalized to 18S rRNA and to 1 for HME. Data are mean ± S.E.M. of three different experiments. **(B)**: AR and actin protein expression by Western blot in wild type HEK, HEK overexpressing AR (HEK-AR), LNCaP, HMEC and PTEC2 line. **(C)**: Cell proliferation of PTEC (black columns) and HUVEC (white columns) after 48 h incubation with 1 μM Sunitinib or Sorafenib in the presence or absence of 10 μM Casodex. Data are the mean ± S.E.M. of a minimum of three independent experiments performed with two PTEC cell lines (PTEC2 and 3) in triplicate. Statistical significance *p < 0.05.

In the attempt to decrease the observed resistance of PTEC to Sorafenib, we evaluated the effect of a combined treatment with the anti-androgen Casodex and the anti-angiogenic drugs. Indeed, functional ARs were described in endothelial cells from benign prostate and prostate cancer [4, 26]. All PTEC lines expressed the AR at mRNA level, being the expression in the PTEC2 line the highest (Figure 5A). The AR expression was confirmed on PTEC2 by means of Western blot, as compared with positive controls such as the AR overexpressing HEK or the prostate LNCaP cells, (Figure 5B).

Cell treatment with Casodex alone (10 μM) significantly reduced cell proliferation of PTEC (Figure 5C) while no effect was observed on HMEC, in accordance with the lower receptor level expression (Figure 5A). The reduction of cell proliferation induced by Sunitinib was not modified by combination with Casodex on both PTEC and HMEC. Interestingly, addition of Casodex was able to counteract the resistance of PTEC to Sorafenib (Figure 5C). No additional effect on reduction of proliferation was observed on HMEC (Figure 5C).

**Figure 6:**
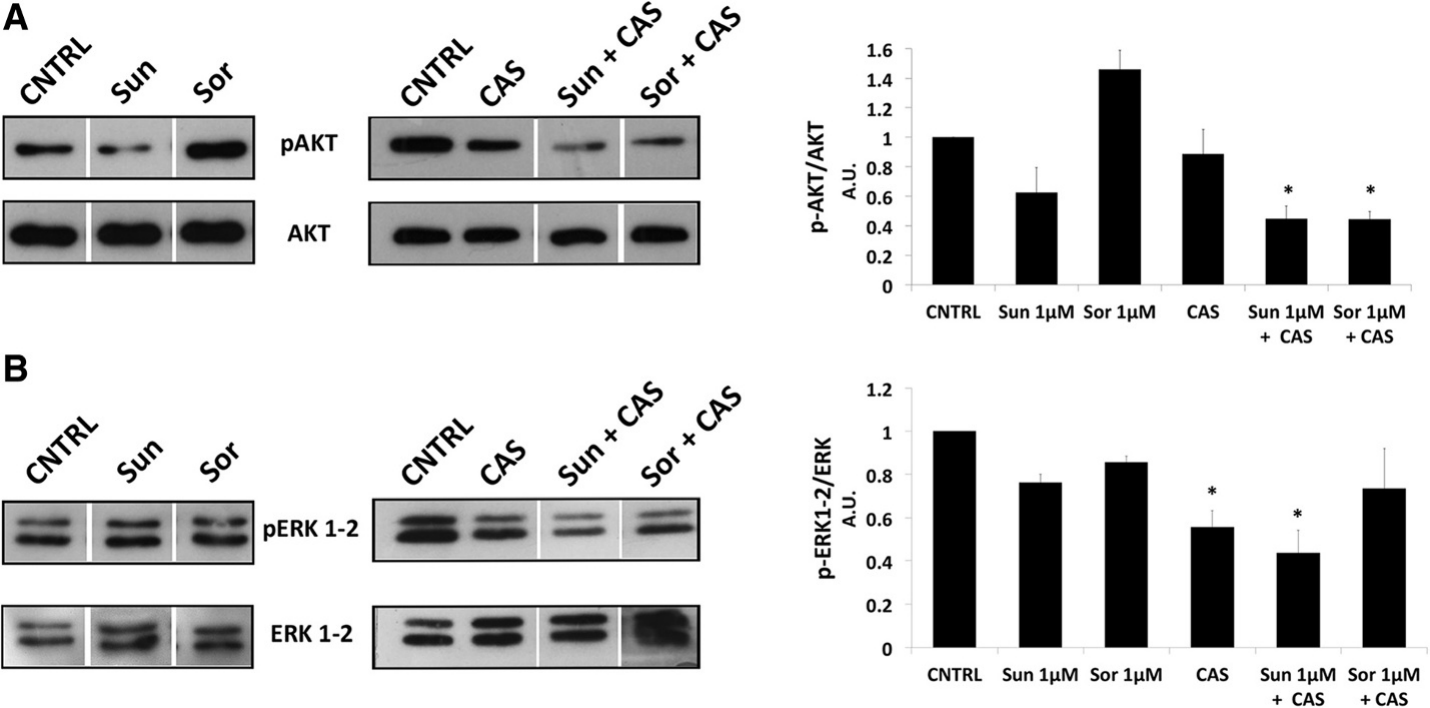
**Effect of Sorafenib and Sunitinib on Akt and ERK1-2 phosphorylation.** Western blot analysis showing basal Akt and phosphorylated Akt (Ser473) (p-AKT) **(A)** or basal ERK1/2 and phosphorylated ERK1/2 (Thr202/Tyr204) (pERK1-2) **(B)** in PTEC2 after 2’ treatment with 1 μM Sunitinib and 1 μM Sorafenib in the presence or absence of 10 μM Casodex. The relative expression of phospho-Akt or phospho-pERK1/2 were normalized to basal Akt or ERK1/2. Values are expressed as mean ± S.E.M. relative to the control of three independent experiments performed with three PTEC2 cell lysate. Statistical significance *p < 0.05.

We subsequently analyzed the signal transduction mechanism involved in PTEC resistance and in its rescue by Casodex. For this purpose, we first tested the effect of both anti-angiogenic drugs on Akt and p44/42MAPK (ERK1/2) activation, important signaling pathways in the effect of anti-angiogenic drugs [27–29]. Sunitinib treatment reduced Akt phosphorylation (Ser473) whereas Sorafenib did not promote any effect (Figure 6A). The inhibitory effect was observed at early times (2 minutes), and quickly reverted by longer treatments (5, 10 and 30 minutes, data not shown). On the other hand, p44/42MAPK (ERK1/2) phosphorylation (Thr202/Tyr204) was not affected by Sorafenib and only slightly by Sunitinib (Figure 6B). This pattern did not change for longer drug treatments (data not shown).

Finally, we evaluated the effect of the combination of Casodex and anti-angiogenic drugs on the intracellular signal transduction pattern observed in PTEC. Cell treatment with Casodex alone did not decrease Akt phosphorylation while a marked effect was detected on p44/42MAPK (ERK1/2) phosphorylation (Figure 6C). When cells were treated with Casodex and Sunitinib, Akt phosphorylation was farther reduced as compared to Sunitinib alone. Interestingly, the combined treatment of Casodex and Sorafenib was able to strongly inhibit Akt phosphorylation in respect to Sorafenib alone. On the other hand, the effect observed with combined treatments of both anti-angiogenic drugs and Casodex maintained the inhibitory phosphorylation effect of the Casodex alone. We can therefore speculate that the Akt intracellular pathway plays a role in the observed resistance of PTEC to Sorafenib. The inhibition of Akt phosphorylation by the combined treatment of Casodex and Sorafenib can therefore explain the rescue observed on cell proliferation.

## Discussion

Taken together, the results of this study show a different sensitivity of endothelial cells isolated from prostate tumors to the anti-angiogenic drugs Sunitinib and Sorafenib. Whereas normal endothelial cells showed similar responses to both drugs in term of proliferation, survival and motility, PTEC were affected by Sunitinib whereas they were more resistant to Sorafenib. However, combined treatment with the anti-androgen Casodex was able to enhance the susceptibility of PTEC to Sorafenib likely trough inhibition of the Akt intracellular pathway.

Several evidences of the literature showed that TEC present in different tumors, including prostate carcinoma, are different from normal endothelial cells at genetic, epigenetic and functional levels [9, 17, 30, 31]. In particular, recent studies of transcriptome and methylome analysis of endothelial cells from healthy or patients affected by prostate cancer showed a wide spectrum of differences in gene expression and methylation patterns in endothelial cells between malignant and normal prostate tissues [30, 31]. In addition, murine endothelial cells from spontaneous prostate tumors were reported to display a mesenchymal differentiative ability [17]. In the present study we isolated and cultured endothelial cells from prostate tumors from patients without androgenic ablation therapy. PTEC were able to migrate, organize in capillary-like structures *in vitro* and in vessel structures *in vivo*, connected with the mouse vasculature, indicating their endothelial phenotype. As previously reported [26], PTEC expressed higher AR levels than normal endothelial cells indicating the persistence of the phenotype of origin. The ability of TEC to organize into functional vessels *in vivo* has been previously described to be characteristic of TEC isolated from human tumors, at variance with HUVEC that undergo apoptosis [10, 17, 18].

PTEC may therefore represent a suitable model to assess the response to anti-angiogenic drugs and the related cell signal mechanisms. Indeed, although the results of anti-angiogenic therapy in preclinical models of prostate cancer provided promising results, some discrepancy between these data and those obtained in clinical trials were observed [32]. In particular, monotherapy treatment of patients with advanced prostate cancer partially failed the endpoints [27, 33–36]. A phase III study comparing Sunitinib versus placebo showed a progression free survival but not an overall survival improvement in Sunitinib treated patients, although phase II studies showed a PSA decline in plasma [33, 37]. Phase II studies with Sorafenib showed a regression of metastases but not PSA decline [34, 36]. Is therefore evident that additional knowledge on endothelial characteristics in prostate cancer is required.

In the present study, we showed that both drugs had a cytotoxic effect on normal endothelial cells with a similar IC50 at 48 hour around 1.5 μM. This is in line with previous observations showing that both Sorafenib and Sunitinib are well known inhibitors of pro-angiogenic functions in normal endothelial cells [16, 38]. Accordingly, Sunitinib induced a dose dependent reduction of proliferation and survival in PTEC as well as in HUVEC and HMEC. In contrast, Sorafenib only partially affected PTEC proliferation and survival. Both drugs slightly reduced cell motility, with a consistent lower effect of Sorafenib. In addition, Sunitinib, but not Sorafenib, affected tubulogenesis in both HUVEC and PTEC. As Sunitinib and Sorafenib share common targets [5, 6], the differential effect of these drugs on PTEC appears unexpected and of interest.

Studies comparing the in vitro activity of Sorafenib and Sunitib on endothelial cells are limited in the literature. A similar effect of the drugs on cell viability was previously reported in neuroblastoma and corneal epithelial cells [39, 40]. At variance, our results, showing that PTEC were less sensible to Sorafenib than to Sunitinib, are in line with the reported resistance to Sorafenib of endothelial cell from hepatocellular carcinoma [16].

As PTEC only expressed the VEGFRs, and not other surface targets of Sunitinib and Sorafenib, such as PDGFRβ and cKIT, we reasoned that the differential response to these drugs could depend on a differential effect on VEGFR2 phosphorylation [6]. However, VEGFR2 phosphorylation was inhibited by both drugs, excluding this possibility. On the other hand, these drugs were reported to affect different intracellular pathways, including the Ras/Raf/MEK/ERK, JAK/STAT and the PI3K/AKT pathways [27–29]. It is conceivable that the increased resistance to Sorafenib observed in PTEC may be due to its activity on intracellular pathways differentially activated in normal and tumor endothelial cells, as reported [10, 41]. In PTEC, we observed that Sorafenib and Sunitinib treatments differentially modulated Akt phosphorylation, as no inhibitory effect of Sorafenib was observed on Akt activation. These data correlate with the functional resistance to the effect of Sorafenib observed on PTEC behavior *in vitro*. On the contrary, no major effect of these drugs was observed on the p44/42MAPK (ERK1/2) pathway. In this regard, Kharazhia and co-workers recently described an increased Sorafenib resistance of highly-metastatic compared with non-metastatic prostate cancer cells which was due to constitutively active PI3K/AKT pathway targeted by Sorafenib [29].

Due to the important role of ARs in sustaining prostate cancer progression, second line hormone therapy is frequently employed in prostate cancer to target persistent AR activation. In addition, AR has been described to play a role in the regulation of endothelial cell proliferation [26]. Moreover, the association of Sorafenib with anti-androgen therapy (Casodex) in a recent phase II clinical trial induced PSA decline and stable disease [42], improving the effect of the anti-angiogenic monotherapy. Accordingly, our results combining Sorafenib and Casodex successfully overcame the PTEC resistance to Sorafenib both at the functional level and on the Akt pathway activation. Therefore, it can be inferred that the resistance to Sorafenib treatment involves the Akt pathway; which is in turn affected by the combined treatment with Casodex.

## Conclusions

In conclusion, the results of the present study clearly demonstrate a resistant behavior of endothelial cells isolated from prostate cancer to specific anti-angiogenic drugs compared to normal endothelial cells. Indeed, it appears that TEC are more appropriate for studying angiogenesis mechanisms of tumors and exploring antiangiogenic drugs, compared with normal endothelial cells. Finally, strategies to combine multi-targeted kinase inhibitors with hormonal therapies may be of interest in the context of prostate cancer.

## Electronic supplementary material

Additional file 1: Figure S1: Cytotoxicity resistance, proliferation and tubulogenesis of Sunitinib and Sorafenib treated PTEC and HMEC. (A and B): Cell survival and proliferation of PTEC (black columns) and HMEC (white columns) after 48 h incubation with 1 μM Sunitinib or Sorafenib. Sorafenib significantly affected HMEC, but not PTEC. Cytotoxicity was detected as MTT assay, proliferation as BrdU assay. (C): Capillary-like organization of PTEC and HMEC. Cells were seeded on Matrigel and observed at different times points. A decrease in tube formation was observed for both PTEC (black columns) and normal HMEC (white columns) after treatment with 2.5 μM Sunitinib, while Sorafenib (2.5 μM) only affected tubule formation in HMEC. Data are the mean ± S.E.M. of a minimum of three independent experiments in triplicate. Statistical significance *p<0.05. (PDF 180 KB)
