# Supplementary material for: Differential sensitivity of prostate tumor derived endothelial cells to sorafenib and sunitinib
Source: BMC Cancer. 2014 Dec 12;14:939. doi: 10.1186/1471-2407-14-939 (PMC4295225; doi:10.1186/1471-2407-14-939)
Supplement: Supplementary file 1 — Additional file 1: Figure S1: Cytotoxicity resistance, proliferation and tubulogenesis of Sunitinib and Sorafenib treated PTEC and HMEC. (A and B): Cell survival and proliferation of PTEC (black columns) and HMEC (white columns) after 48 h incubation with 1 μM Sunitinib or Sorafenib. Sorafenib significantly affected HMEC, but not PTEC. Cytotoxicity was detected as MTT assay, proliferation as BrdU assay. (C): Capillary-like organization of PTEC and HMEC. Cells were seeded on Matrigel and observed at different times points. A decrease in tube formation was observed for both PTEC (black columns) and normal HMEC (white columns) after treatment with 2.5 μM Sunitinib, while Sorafenib (2.5 μM) only affected tubule formation in HMEC. Data are the mean ± S.E.M. of a minimum of three independent experiments in triplicate. Statistical significance *p<0.05. (PDF 180 KB) [file 12885_2014_5098_MOESM1_ESM.pdf]

Supplementary Figure 1. Cytotoxicity, proliferation and tubulogenesis of Sunitinib and Sorafenib treated PTEC and HMEC.

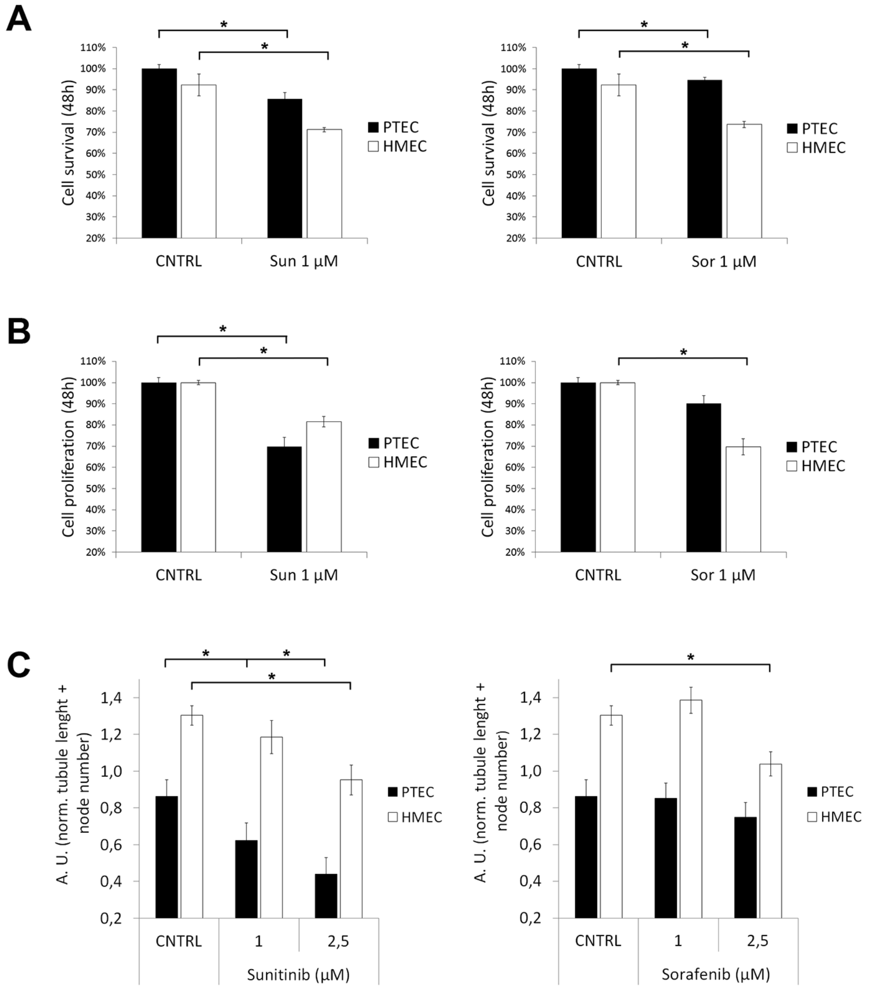

Supplementary Figure 1. Cytotoxicity resistance, proliferation and tubulogenesis of Sunitinib and Sorafenib treated PTEC and HMEC. **(A and B)**: Cell survival and proliferation of PTEC (black columns) and HMEC (white columns) after 48 h incubation with 1 µM Sunitinib or Sorafenib. Sorafenib significantly affected HMEC, but not PTEC. Cytotoxicity was detected as MTT assay, proliferation as BrdU assay. **(C):** Capillary-like organization of PTEC and HMEC. Cells were seeded on Matrigel and observed at different times points. A decrease in tube formation was observed for both PTEC (black columns) and normal HMEC (white columns) after treatment with 2.5 µM Sunitinib, while Sorafenib (2.5 µM) only affected tubule formation in HMEC. Data are the mean ± S.E.M. of a minimum of three independent experiments in triplicate. Statistical significance * $p<0.05$.
